# Typology of Agricultural Land Systems in Germany to Support Tailoring of Agri-Environmental Schemes to Reduce Pressures on Farmland Biodiversity

**DOI:** 10.1007/s00267-026-02490-5

**Published:** 2026-06-22

**Authors:** Martin Pingel, Diana Sietz, Norbert Röder, Sebastian Klimek, Burkhard Golla

**Affiliations:** 1 Julius Kühn Institute (JKI) – Federal Research Centre for Cultivated Plants, Institute for Strategies and Technology Assessment, Kleinmachnow, Germany; 2 Thünen Institute of Biodiversity, Braunschweig, Germany; 3 Thünen Institute of Rural Studies, Braunschweig, Germany

## Abstract

Farmland biodiversity and associated ecosystem services are threatened by the expansion and intensification of agriculture. Despite substantial efforts, European conservation policies have largely failed to halt or reverse the decline of farmland biodiversity. To address the pressures associated with agricultural intensification more effectively, conservation measures - such as agri-environmental schemes – need to be tailored based on spatially explicit land system classifications that reflect the interdependencies between agricultural land use and biodiversity. In this study, we present a spatially explicit typology of agricultural land systems in Germany offering a meaningful generalization of the country’s agricultural landscapes to inform and support the tailoring of agri-environmental schemes. We applied a cluster analysis based on indicators of land cover, landscape structure, land-use intensity of different production systems, as well as biophysical indicators, such as climate and topography. Experts from agriculture, conservation, and administration sectors contributed to refining both the indicator selection and the clustering results. Clustering revealed eight distinct agricultural land system types in Germany, including arable cash crop-dominated, animal husbandry-dominated, and mosaic land-use agricultural land system types. Each type represents a characteristic combination of indicators, offering key insights into the specific pressures affecting farmland biodiversity. Drawing on this typology, we discuss entry points for tailoring agri-environmental schemes to reduce land system-specific pressures on biodiversity. This typology has the potential to increase the ecological effectiveness of agri-environmental schemes by tailoring them to land system-specific pressures. Moreover, the methodological approach can be adapted to other countries or scaled beyond the national level.

## Introduction

Worldwide, biodiversity and associated ecosystem services in agricultural landscapes are threatened by the expansion and intensification of agriculture (Sánchez-Bayo and Wyckhuys 2019; Rigal et al. 2023). Agricultural intensification involves increases in inputs such as pesticides or fertilizers at the field and farm scale with direct negative effects on farmland biodiversity (Kleijn et al. 2009; Nicholson et al. 2024). Other inputs such as fodder imports to feed livestock animals in housing systems result in high rates of nitrogen deposition from manure (de Vries et al. 2021) that negatively affect biodiversity on farmland and non-farmland habitats (Bobbink et al. 2010). At landscape scales, agricultural intensification leads to landscape simplification and homogenization through the decrease of semi-natural habitats, crop specialization, and field enlargement (Emmerson et al. 2016), thereby contributing to biodiversity losses of many different taxa (Sirami et al. 2019).

The European Union (EU) has recently implemented the Nature Restoration Regulation (NRR) with the aim of halting and reversing biodiversity losses (European Parliament and European Council 2024). The regulation requires member states, among others, to enhance biodiversity in agricultural landscapes and to achieve an increasing trend in certain key biodiversity indicators at national level (Article 11) by implementing effective restoration measures. The European Union’s Common Agricultural Policy (CAP) is a key tool in reaching these biodiversity targets (European Court of Auditors 2020). Within the recent CAP, the voluntary eco-schemes and environmental, climate-related, and other management commitments (ENVCLIM) aim at mitigating the negative effects of agricultural intensification on farmland biodiversity and associated ecosystem services by providing payments to farmers for implementing more biodiversity-friendly farming practices (Pe’er et al. 2022; Röder et al. 2024). These agri-environmental schemes (AES) encompass a range of on-field measures such as reducing land-use intensity by restricting livestock stocking rates or agrochemical inputs, and off-field measures, such as promoting the maintenance or creation of semi-natural habitats (e.g., hedges, fallows or wildflower strips). However, despite some positive effects (Batáry et al. 2015), AES did not reverse negative trends of farmland biodiversity (Kleijn et al. 2011; European Court of Auditors 2020). Their limited ecological effectiveness has been attributed to, amongst other reasons, the poor uptake of AES by farmers (e. g., Brown et al. 2021; Pe’er et al. 2022), the absence of spatial coordination at landscape scales (Nguyen et al. 2022), and the poor tailoring of AES to effectively address environmental pressures from agriculture at the landscape level (Uthes et al. 2010; Klimek et al. 2014). Tailoring, in this context, refers to selecting and prioritizing AES to address specific properties of agricultural land systems (Sietz et al. 2022). However, the tailoring of AES to effectively mitigate pressures on biodiversity is challenging due the lack of spatial explicit land system classifications that capture the key environmental pressures arising from the interdependencies between agricultural land-use and biodiversity.

Given the interdependencies between land-use and the environment, various processes recur across regions, motivating extensive pattern analysis (Ellis and Ramankutty 2008; Dou et al. 2021). Patterns depict functional similarities emerging between agriculture, biodiversity, climate and other social-ecological dynamics (Sietz and Neudert 2022). Pattern recognition is fundamental in the portfolio of methods used in archetype analysis (Sietz et al. 2019), which identifies recurring configurations of factors and processes determining the (un)sustainability of social-ecological systems and land governance. Pattern or archetype analysis bridges the local or farm scale, where AES are implemented by farmers, and the national scale, where such schemes are designed within national CAP strategic plans.

Several European classifications have been developed with the purpose to inform and support tailoring of agri-environmental policy interventions (van der Zanden et al. 2016; Levers et al. 2018; Rega et al. 2020) and support the tailoring of interventions (Beckmann et al. 2022). Focusing on biophysical variables, Beckmann et al. (2022) identified archetypes of agri-environmental potential. Levers et al. (2018) and Rega et al. (2020) developed classifications using data on land-use types and land-use intensity, which resulted in land system archetypes or crop management systems that differentiate land system classes of low, medium and high intensities. The typology developed by van der Zanden et al. (2016) also included landscape structure, which contributed to a more nuanced map not only distinguishing agricultural systems along the intensity gradient but also differentiating them by their landscape heterogeneity. While European classifications provide valuable information at a continental scale (e. g., Rega et al. 2020), which could inform policy tailoring at the EU level, national AES should be tailored based on classifications that address the unique characteristics of agricultural land systems at the subnational and regional level. However, spatial classifications entirely covering Germany were solely built on environmental variables, such as climate and soil (Schröder and Schmidt 2001; Ließ 2022). Other typologies in Germany are not spatially explicit, though capturing important characteristics of farms, including social characteristics, farm structure and management, and the biophysical context (Weltin et al. 2017; Graskemper et al. 2021). This limits our understanding of the spatial heterogeneity of agricultural landscapes and hampers the tailoring of AES to effectively reduce pressures on biodiversity in agricultural regions in Germany.

Agricultural land system typologies should capture all dimensions of agricultural intensification, including land-use/land cover, landscape structure and the intensity of agricultural land-use (Firbank et al. 2008), since these dimensions moderate the ecological effectiveness of AES (Kleijn et al. 2011; Marja et al. 2019; Meier et al. 2022). Both components of landscape structure, composition and configuration, exhibit strong influences on farmland biodiversity (e. g., Ekroos et al. 2010). The configuration of landscapes (e.g., size, shape and spatial arrangement of land-use patches), in addition to their composition (proportion of land-use types), is a key factor in determining biodiversity and associated ecosystem services in agricultural landscapes (Martin et al. 2019). Recent evidence suggests that indicators of landscape structure, such as field size, can even have larger effects on farmland biodiversity than farming practices (Martin et al. 2020). Land-use intensity refers to the intensity of agricultural management at the field level (Dullinger et al. 2021). Negative effects on biodiversity arising from high land-use intensity are connected to high rates of agrochemical inputs, such as pesticides and mineral fertilizers (Kleijn et al. 2009; Nicholson et al. 2024), or high rates of nitrogen deposition as a consequence of high livestock densities and large quantities of manure (Bobbink et al. 2010; de Vries et al. 2021). For characterizing land-use intensity, variable input costs per hectare have been considered a suitable indicator because they aggregate a variety of inputs, which are linked to farming practices, including pesticides, mineral fertilizers, machinery-use, and fodder imports for livestock (Teillard et al. 2012). The variable input cost indicator in conjunction with indicators describing landscape structure can provide a better understanding on the dominant pressures on farmland biodiversity (Lorel et al. 2019).

Here, we present a spatially-explicit typology of agricultural land systems (ALS) in Germany. We conceptualize ALS as terrestrial social-ecological systems in which human and environmental systems interact through agricultural land-use (Meyfroidt et al. 2022). Germany covers wide gradients in biophysical and agri-environmental conditions and therefore could serve as a suitable role model for transferring results to other European contexts. We applied cluster analysis as a prominent pattern recognition method suited to identify patterns in high-dimensional data spaces based on similarities. The underlying indicators were refined by consultations of experts from the fields of agriculture, nature conservation, science, and administration. The characteristic patterns of indicator values of ALS types were then interpreted in terms of underlying processes that result in agriculture-related pressures on farmland biodiversity. We finally elaborated entry points for tailoring of AES to effectively reduce pressures on biodiversity.

## Methods

### Methodological Overview

We applied a cluster analysis combined with expert consultation to reveal patterns of ALS in Germany. We chose clustering as an unsupervised pattern recognition method (Sietz et al. 2019). Through partitioning data into groups with similar characteristics, clustering serves to identify recurring combinations of factors and processes that affect farmland biodiversity.

First, we compiled a set of indicators representing land cover, landscape structure, land-use intensity, and biophysical parameters as inputs for cluster analysis (Supplementary Table 1). Indicators were selected based on their ability to reflect the diversity of agricultural landscapes and biodiversity-land use interactions. Additionally, the underlying data needed to be comprehensive and of high quality at the national level. Using the initial indicator set, a cluster analysis was conducted resulting in a draft of the typology (Supplementary Fig. 1). All indicators were resampled to a hexagonal grid with a resolution of 1 km^2^ (Perić et al. 2022). While both rectangular and hexagonal grids have its benefits a hexagonal grid was chosen due to its greater clarity in visualization, and to facilitate further applicability of the ALS typology in future research, such as when analyzing connectivity and neighborhood analysis (Birch et al. 2007).

In February 2021, fifteen experts from across Germany participated in a half-day workshop. All participants had expertise in agriculture and its impacts on biodiversity, representing the fields of agriculture, nature conservation, science, and administration. This expert workshop aimed at a) refining the set of indicators considering their relevance for the study objectives, as well as data completeness and availability, and b) validating the typology considering its applicability for tailoring AES to reduce land system-specific pressures on biodiversity.

Reviewing the initial indicator set, the experts raised concerns regarding some indicators and suggested alternatives (Supplementary Table 1): For example, they recommended excluding the indicator density of hedges due to concerns about data accuracy. Instead, they suggested including indicators for farmland patch size and farmland edge density as proxies for configurational landscape heterogeneity. They also recommended selecting appropriate climate indicators based on a broader range of parameters, including monthly rather than annual temperature and precipitation data. Moreover, the experts raised concerns about the inclusion of forest cover. Yet, forests are highly relevant as they provide habitats for ecotone species and often exhibit dynamic interactions along the boundaries between forests and agricultural land. In addition, they suggested to include livestock density as an indicator of land-use intensity. However, comparable indicators for the intensity of arable farming or permanent crops are not available at the local level (local administrative units, LAU 2). For this reason, data on the variable costs of livestock farming were used as a more integrative indicator for land-use intensity, as comparable integrated cost indicators for plant-based production systems are available.

We followed these expert’s recommendations, updated the final set of indicators (see section “Indicators”, Table 1, and Supplementary Table 1), and re-run the cluster analysis as the next step of the methodological workflow. In this cluster analysis, the optimal number of clusters was determined by evaluating internal validity (Piemontese et al. 2022) through stability considerations (see section “Cluster analysis”). With eight clusters identified, a new draft of the typology was produced, for continuing the discussion with the experts in a second workshop. The experts agreed that the typology and the regional distribution of ALS types were plausible and that the ALS types adequately reflect the diversity of agricultural landscapes, the interactions between agricultural land-use and biodiversity, and the main agricultural-driven pressures on biodiversity.

**Table 1:** Overview of indicators used for cluster analysis to classify agricultural land systems.

Domain	Name (abbreviation)	Unit	Temporal reference	Source
Land cover	Arable land	Share of grid cell area (%)	2016	ATKIS Basic Digital Landscape Model (DLM) (BKG 2017)
	Permanent crops and horticulture	Share of grid cell area (%)	2016	ATKIS Basic DLM (BKG 2017)
	Grassland	Share of grid cell area (%)	2016	ATKIS Basic DLM (BKG 2017)
	Forests	Share of grid cell area (%)	2016	ATKIS Basic DLM (BKG 2017)
	Settlements	Share of grid cell area (%)	2016	ATKIS Basic DLM (BKG 2017)
	Semi-natural habitats	Share of grid cell area (%)	2016	ATKIS Basic DLM (BKG 2017)
Landscape structure	Shannon diversity of land cover classes	unitless	2016	ATKIS Basic DLM (BKG 2017)
	Farmland^1^ edge density	m/ha	2016	ATKIS Basic DLM (BKG 2017)
	Farmland^1^ patch size	ha	2016	ATKIS Basic DLM (BKG 2017)
Land-use intensity	Variable costs for arable cash crops	Euro/ha	2014–2018	Röder et al. 2022
	Variable costs for permanent crops and horticulture	Euro/ha	2014–2018	Röder et al. 2022
	Variable costs for pig and poultry farming	Euro/ha	2014–2018	Röder et al. 2022
	Variable costs for dairy farming and intensive beef fattening	Euro/ha	2014–2018	Röder et al. 2022
	Variable costs for extensive livestock farming	Euro/ha	2014–2018	Röder et al. 2022
Biophysical parameters	Mean annual temperature	°C	2000–2019	DWD 2022
	Temperature seasonality	°C	2000–2019	DWD 2022
	Evapotranspiration March	mm	2000–2019	DWD 2022
	Relief heterogeneity	unitless	-	Digital Elevation Model 200 m (BKG 2019)

^1^Farmland refers to land cover classes arable land, grassland, and permanent crops and horticulture.

### Indicators

#### Land cover and landscape structure

For land cover indicators, objects of the Basic Digital Landscape Model of the German Official Topographic Cartographic Information System of Germany (ATKIS Basic DLM, BKG 2017) were assigned to the following six land cover classes (see Supplementary Table 2: coarse thematic resolution): arable land, permanent crops and horticulture, grassland, forests, settlements (built-up areas for residential, industrial and commercial use), semi-natural habitats (heaths, bogs, swamps, wetlands, small woody habitats, wasteland). For each grid cell, the share of each land cover classes was calculated. The ATKIS Basic DLM is a nationwide, object-oriented vector geodatabase describing topographic features of Germany. It is maintained by the surveying authorities of the German federal states and based on authoritative topographic surveys and maps. The dataset is intended for regional to national analyses and corresponds to a nominal scale range of approximately 1:10 000 to 1:25 000. It serves as a primary reference for numerous scientific disciplines, particularly landscape ecology and environmental monitoring (e.g., Eichler et al. 2022). It is also an essential data source for policy advising (e.g., Golla et al. 2023).

For landscape diversity, the Shannon diversity as a metric for landscape composition (Firbank et al. 2008) was derived for each grid cell based on the shares of 15 land cover classes taken from ATKIS Basic DLM objects using the function ‘diversity’ from package ‘vegan’ version 2.5–7 (Oksanen et al. 2019) of the statistical software R (R Core Team 2021). The 15 land cover classes were arable land, vineyards, fruit orchards, orchard meadows, horticulture, hops, tree-nurseries, grassland, forests, settlements, heaths, bogs, swamps and wetlands, small woody habitats, wasteland (see also Supplementary Table 2: fine thematic resolution). Calculation of farmland edge density and mean farmland patch size was based on land cover objects classified as farmland (arable land, grassland, permanent crops, and horticulture). Farmland edge density was obtained by summing up the total length (m) of all farmland polygon edges within each grid cell. The length was corrected for the size of the grid cell and the coverage of the ATKIS Basic DLM to obtain values in the unit m/ha. Mean farmland patch size was calculated by averaging sizes of all farmland polygons lying within each grid cell and those overlapping with its boundaries.

#### Land-use intensity

To integrate several categories of agricultural inputs into one continuous land-use intensity indicator, we used the variable costs of agricultural production per hectare (variable costs hereafter), which is defined as “the ratio between the sum of different categories of input costs and the total Utilized Agricultural Area” (Teillard et al. 2012). To calculate the variable costs indicator, variable costs for operating and production resources were compiled using national data sources (BLfL 2020; KTBL 2020). The aforementioned costs were differentiated at the regional level (NUTS-Level 2) and were further regionalized at the spatial resolution of counties (NUTS-Level 3). This differentiation is based on the average natural yields of main crops (Federal Statistical Office of Germany 2025) and milk yield per dairy cow (BLE 2022) for the years 2014 to 2018. County-level data were downscaled using data on the distribution of the main agricultural production systems at the municipality level (Local Administrative Units, LAU 2) based on land use (including livestock husbandry) and land cover data for 2016 (Neuenfeldt et al. 2020). We used the average of the yearly data for the period 2014 - 2018 to account for inter-annual variation of prices and yields (Teillard et al. 2012). A detailed description of cost calculations is provided in Röder et al. (2022). For this study, we did not consider labour costs.

Because the magnitude of variable costs and categories of agricultural inputs differ widely between production systems, we differentiated the variable costs for the following major production systems based on the farming typology used in the farm accountancy data network (EC 2014):

1. Arable cash crops (including cereals, legumes, potatoes, sugar beets, field vegetables, oil rape seed, and forage crops).
2. Permanent crops and horticulture (including fruits, grapevines, hops, horticultural products, and short rotation forestry).
3. Pig and poultry farming.
4. Dairy farming and intensive beef fattening (including dairy cows, bulls, and corn for biogas production). Costs associated to the production of heifers, grass, hey and silage were assigned to this system.
5. Extensive livestock farming (including pasture management of suckler cows, sheep, goats, and horses).

Costs associated with the production of heifers, grass, hay and silage were proportionally allocated to the production systems (4) and (5) based on the respective share of dairy and suckler cows in the local cow stock. An agro-economic perspective on the use of variable costs as land-use intensity indicator and additional information on input categories of livestock production systems are given in the Supplementary Methods 1.

#### Biophysical indicators

For climate indicators, we analyzed 76 climate variables averaged over the period of 2000 to 2019 (DWD 2022) using principal component analysis (PCA) to reduce the dataset to three uncorrelated indicators representing the main climate gradients in Germany. PCA was done using the R-packages FactoMinerR version 2.4 (Lê et al. 2008) and factoextra version 10.0.7 (Kassambara and Mundt 2020).

The first principal component (Dim1) was strongly correlated with temperature variables (Supplementary Fig. 2a). Among the variables contributing most to the variance of the first component was mean annual temperature (Supplementary Fig 2a), which we selected as climate indicator representing temperature gradients in Germany. The second principal component (Dim2) was primarily associated with temperature seasonality (Supplementary Figs. 2a, 3b), with the temperature difference between of monthly means of July and January selected as the corresponding indicator. For the third principal component (Dim3, Supplementary Figs. 2b, 3c), potential evapotranspiration in March had the highest contribution (Supplementary Fig. 3c). Evapotranspiration is a modelled variable that integrates multiple factors including temperature and precipitation (DWD Climate Data Center 2018). As it effectively describes the water regime for cultivated plants, it was included as a climate indicator. For a detailed description of the derivation of climate indicators, refer to the Supplementary Methods 2.

To describe relief heterogeneity, the Terrain Ruggedness Index (Riley et al. 1999) was calculated based on the digital terrain model at a resolution of 200 m (BKG 2019) using the RGDAL function in QGIS version 3.4.15 (QGIS.org 2022). For a summary of all indicators, see Supplementary Table 3.

#### Data processing

Grid cells with missing data or those that contained less than in total 5% of arable land, grassland, permanent crops and horticulture, and semi-natural habitats were excluded from the cluster analysis (Supplementary Table 4, Supplementary Fig. 4). The threshold of 5% for agricultural area was empirically derived and ensured that forest and urban grid cells were excluded, while marginal agricultural land-use areas at the edges of forests and urban areas were retained. The final data set encompassed 315,318 grid cells. We checked correlations between indicators using Spearman rank correlation. All correlation coefficient were below |0.7| indicating only moderate collinearity between our indicators (Supplementary Fig. 5). Extreme indicator values, which skew the data distribution, were interpreted as outliers. To de-skew respective data, we replaced outliers, which we defined as data above the 99^th^ percentile, with the values at the respective 99^th^ percentile. This procedure was applied to mean farmland patch size and variable costs of production systems except for arable cash crops (Supplementary Table 3). To enable comparison, all indicators were z-standardized. All geospatial and clustering tasks were conducted using R version 4.1.02. and newer (R Core Team 2021) if not indicated otherwise.

### Cluster analysis

Clustering was performed using k-medians, a partitioning algorithm implemented in the R package flexclust (Leisch 2006), reflecting the non-normal distribution of some indicators. Based on stability considerations (Ben-Hur et al. 2002; Sietz et al. 2017), we determined the optimal number of clusters using the ‘stepFlexclust’ function implemented in flexclust. We considered cluster numbers from k = 2–20, performed 100 cluster runs for each of these cluster numbers and retained the best partition among all repetitions based on minimum within-cluster variance. The best partition for each k was compared with 500 independently derived partitions in a pair-wise way using the ‘kcca’ function and the adjusted Rand Index (aRI) (Hubert and Arabie 1985). The aRI served as a metric for cluster stability. The mean aRI of all paired comparisons for each predefined k was used as the basis for determining the optimal cluster number.

In a second half-day workshop in November 2021, the experts reviewed the plausibility of cluster results. In particular, the experts discussed a) the credibility of the patterns of indicator values of ALS types and their spatial distribution (e.g., sufficient regional differentiation, and b) the typology’s applicability and suitability to support tailoring of AES. The participatory approach thus helped to increase the potential typology’s impact, hence strengthening its application validity (Piemontese et al. 2022).

These expert discussions informed the decision about the optimal number of clusters among those partitions that showed a high aRI Index (see Supplementary Fig. 6). The clusters, i.e., typical associations of indicator values, were described and named based on the indicator values at the cluster center and the clusters’ spatial distribution.

## Results

**Fig. 1:**
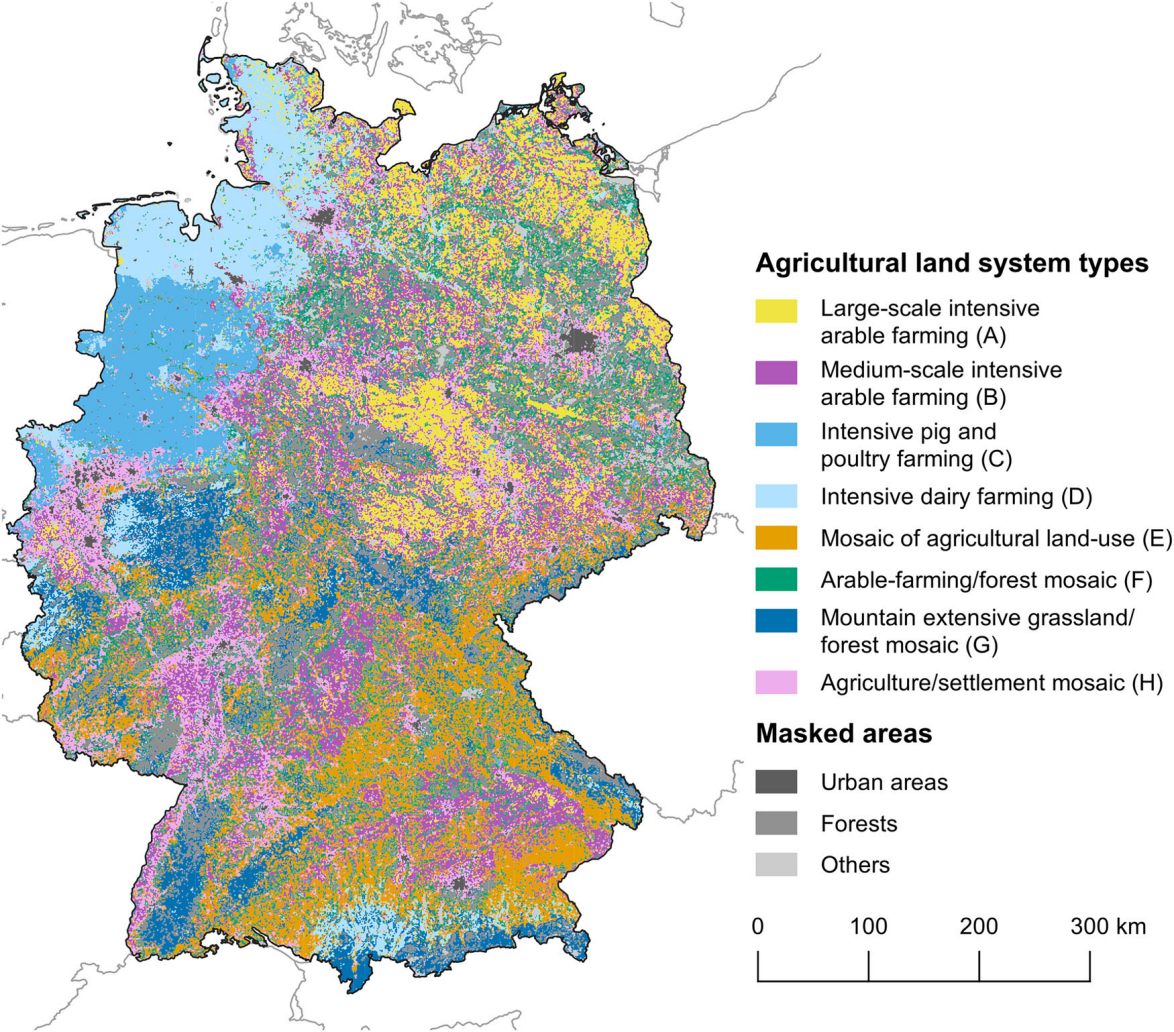
Distribution of agricultural land system (ALS) types (A-H) in Germany. Masked areas (grey-shaded) were not used for cluster analysis. For maps of individual ALS types, see Supplementary Fig. 7. Source of national borders: © EuroGeographics

The application of cluster analysis resulted in eight distinct ALS types (Fig. 1, Table 2, Supplementary Fig. 7). The experts agreed with the typology with regard to the plausibility of the diversity of agricultural landscapes in Germany. The aRI Index showed a plateau of similarly robust partitions for k = 6–12 (see Supplementary Fig. 6).

**Table 2:** Area covered by agricultural land system (ALS) types and respective share of the total agricultural area of Germany

Agricultural land system type		Area (km²)	Area share (%)
A	Large scale intensive arable farming	36,460	11.6
B	Medium-scale intensive arable farming	60,907	19.3
C	Intensive pig and poultry farming	22,982	7.3
D	Intensive dairy farming	30,095	9.5
E	Mosaic of agricultural land use	60,376	19.1
F	Arable-farming/forest mosaic	42,357	13.4
G	Mountain extensive grassland/forest mosaic	30,195	9.6
H	Agriculture/settlement mosaic	31,946	10.1

**Fig. 2:**
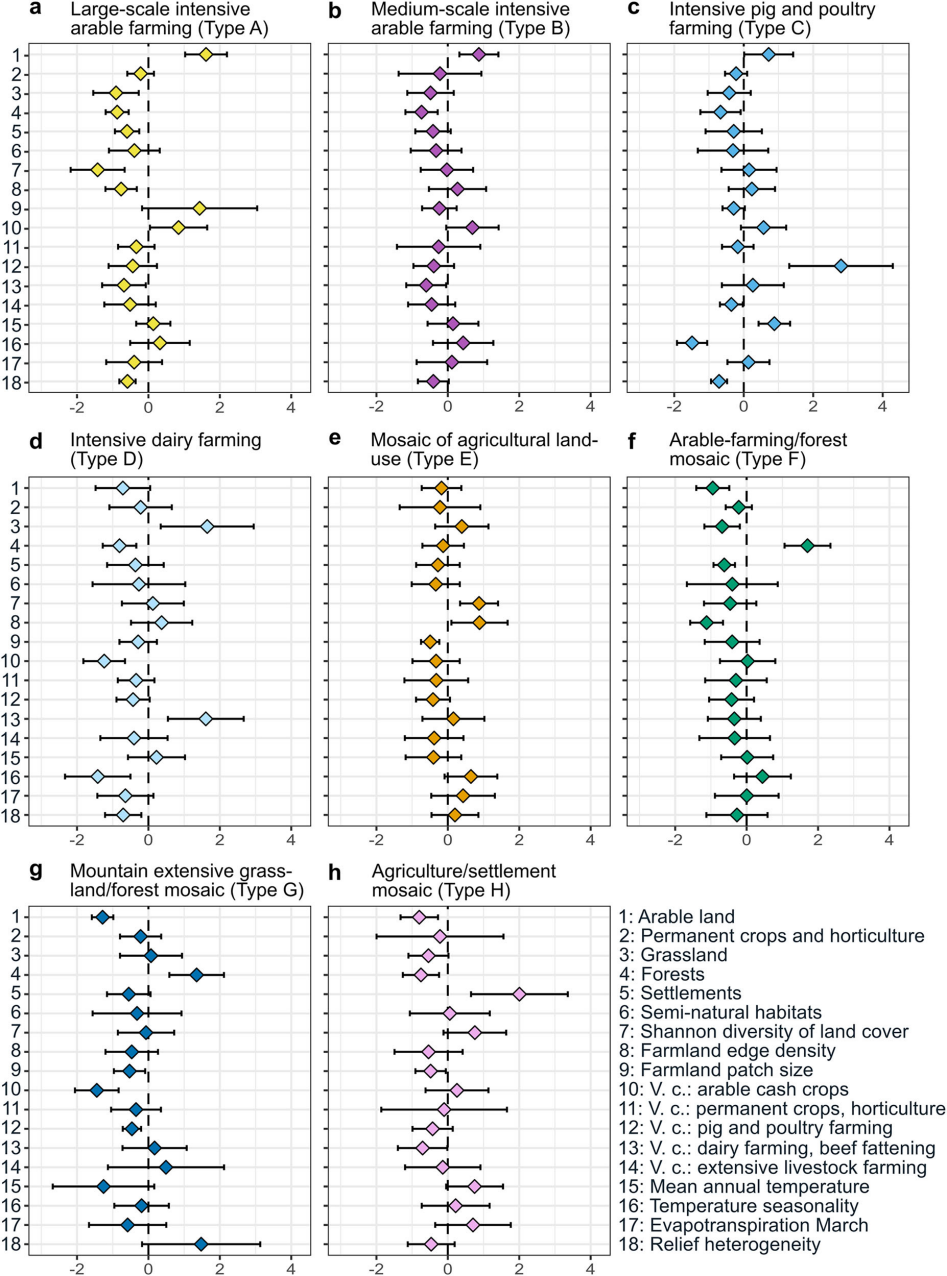
Median values (± standard deviation) of z-score-standardized indicator values within a given cluster for agricultural land system (ALS) types (**a**–**h**). Dashed vertical lines indicate the mean value across all regions included in the analysis. V. c. Variable costs

The ALS types are described by the medians of the standardized indicator values (Fig. 2). Indicator values deviating from the respective national means by at least one standard deviation were considered particularly important for characterizing the ALS types and deriving agricultural pressures on farmland biodiversity. For a summary of all input parameters, see Supplementary Table 5 and Supplementary Fig. 8. To characterize the ALS types, we grouped them based on the variable costs for different production systems and land cover classes, leading to three groups: 1) Arable cash crop-dominated ALS (types A and B), 2) Animal husbandry-dominated ALS (types C, D, and G), and 3) Mosaic land-use and land cover ALS (types E, F, and H).

### Arable cash crop-dominated ALS: types A and B

The ALS types *Large-scale intensive arable farming* (type A) and *Medium-scale intensive arable farming* (type B*)* exhibit a high share of arable land cover, with both medians deviating from the national mean by more than one standard deviation (Fig. 2a, b). Type A shows the highest share of arable land; resulting in low shares of grassland, forests, and settlements, as well as low Shannon diversity of land cover classes. It also features low farmland edge density and the highest farmland patch sizes among all ALS types, indicating structurally simplified landscapes with large-sized agricultural fields. Type B exhibits similar land cover patterns but less pronounced deviations from the national mean. Both types show above-average variable costs for arable cash crops, with type A having the highest values among all ALS types. The indicators for landscape structure are close to the German-wide mean. Animal husbandry plays a minor role in both types. Both types are primarily located in flat regions, as indicated by low relief heterogeneity.

Type A is located in northeastern Germany, particularly in areas with productive Chernozem/black soils and loess soils north of the Harz mountain range, as well as in Bavaria (Danube and Main rivers) and in North Rhine-Westphalia (Fig. 1, Supplementary Fig. 7a). Type B, the largest ALS type by area (Table 2), is more widely distributed with concentrations in northeastern Germany and near the Rhine, Neckar, and Danube rivers (Supplementary Fig. 7b).

### Animal husbandry-dominated ALS: types C, D, and G

In ALS types *Intensive pig and poultry farming* (type C), *Intensive dairy farming* (type D), and *Mountain extensive grassland/forest mosaic* (type G), animal husbandry systems play an important and characteristic role. However, the types strongly differ regarding the composition of land cover, the dominating type of livestock animal and the intensity of the production systems.

Type C shows a land cover compositional pattern similar to type B, presenting high shares of arable land. In contrast, type D exhibits the highest share of grassland, while shares of arable land and forest are below the national average (Fig. 2d). In type G, forest is the dominating land cover, while the values for arable land are the lowest compared to the other ALS types (Fig. 2g, Supplementary Table 5). This pattern overall indicates that the land not covered by forests is mainly used for grasslands in this type. Regarding landscape structure, type C and D show median indicator values close to the German-wide mean. In type G, values for both farmland edge density and farmland patch size are below the national mean.

Land-use intensity indicators differ strongly between types C, D, and G: type C is characterized by the highest variable costs for pig and poultry farming compared to other ALS types, but also values for variable costs of arable cash crop production are above the national mean. Type D is dominated by intensive dairy farming, which is expressed by high variable costs for dairy farming and intensive beef fattening. Variable costs for arable cash crops are below the national average. In regions of type G, non-forest land is used for extensive livestock farming and dairy cattle farming indicating by above-mean values for variable costs for dairy cow farming and beef fattening, and extensive livestock farming, although the means do not deviate strongly from the national mean.

Types C and D are characterized by the Atlantic climate regime indicated by low temperature seasonality. Additionally, the mean annual temperature is relatively high in type C The climate is characterized by low temperature seasonality and low evapotranspiration in March. Both types show below-average values for relief heterogeneity indicating a flat terrain. Type G exhibits highest values of relief heterogeneity and relatively low values for mean annual temperature.

Type C is the smallest ALS type in terms of area (Table 2) and shows a contiguous distribution in Northwest Germany between Bremen and the Rhine-Ruhr-Area (Supplementary Fig. 7c). Type D is geographically divided into several contiguous regions: The largest is located in Northwest Germany at the coast of the North Sea. The Sauerland (Eastern of the Rhine-Ruhr-Area), the foreland of the Alps, and the Eifel Mountains are also characterized by type D (Supplementary Fig. 7d). Type G is restricted to the mountain ranges Rhenish Massif (Sauerland, Eifel Mountains), Thuringian Forest, Ore Mountains, Rhön, Black Forest, Swabian Jura, Southern Bavarian Forest and Bavarian Alps (Supplementary Fig. 7g).

### Mosaic land-use and land cover ALS: types E, F, and H

The *Mosaic of agricultural land-use* (type E) is characterized by land cover composition resembling the national mean (Fig. 2e). The *Arable-farming/forest mosaic* (type F) is dominated by forest, with other land cover classes falling below the national average. The *Agriculture/settlement mosaic* (type H) is distinguished by its high share of settlements (Fig. 2h), while the shares of arable land, grassland, and forest are below the national average.

Type E exhibits the highest Shannon diversity and farmland edge density, as well as the lowest farmland patch size among all ALS types (Supplementary Table 5), indicating the presence of structurally complex landscapes. Type H demonstrates relatively high values for Shannon diversity, while farmland edge density values are comparatively low. Type F exhibits relatively low level of configurational landscape diversity, indicated by low farmland edge density values, similar to the values of the type A.

In terms of land-use intensity, type E and F exhibit no strong deviations from the national mean for any production system. For type H, the variable costs for intensive dairy farming as well as pig and poultry farming are below the national average, indicating that intensive animal husbandry is of lower priority in these regions.

Regarding climate, type E and F shows higher-than-average temperature seasonality indicating a rather continental climate regime. Type H exhibits high values for mean annual temperature and evapotranspiration in March, indicating a relatively warm and dry climate in the corresponding regions. Relief heterogeneity of types E and F resemble the national average, while regions of type H are characterized by rather flat landscapes.

Geographically, type E is mostly limited to the South of Germany, where it is widely distributed and the most important ALS type of the uplands of Bavaria, Baden-Württemberg, Rhineland-Palatinate, Saarland, and Hesse (Supplementary Fig. 7e). It is the second largest ALS type (Table 2). Type F exhibits a scattered pattern and is distributed across Germany, except for the Northwestern part (Supplementary Fig. 7f). A higher density of this type can be observed in the East German federal states Brandenburg and Mecklenburg-Western Pomerania as well as in Western Lower Saxony. Type H is regionally associated with urban centers, metropolitan areas, and, interestingly, with vine-growing regions in the southwest of Germany (Rhine, Palatine, Rhine-Hesse, Moselle). Due to the scattered pattern of urban centers, this type also shows a scattered pattern (Supplementary Fig. 7h).

## Discussion

### Methodological rationale and advantages of the typology of ALS

The combination of a data-driven clustering approach and expert consultation throughout the development of the typology of ALS enabled us to plausibly reflect the diversity of agricultural land systems in Germany, and can help to achieve a broad acceptance of the typology. By integrating expert perspectives from the fields of agriculture, nature conservation, science and administration which are directly linked to the management of ALS and the design of AES programmes, we aimed at ensuring the credibility and acceptance of the typology. The experts agreed that the typology of ALS presents a meaningful generalisation of heterogeneous agricultural land systems in Germany suitable to inform the tailoring of AES.

The typology was based on indicators capturing major drivers of farmland biodiversity, such as land cover, landscape structure, land-use intensity (Firbank et al. 2008). Further, the inclusion of biophysical indicators such as climate and relief, which have a strong influence on land-use intensity (Meier et al. 2022), helped to reveal further insights into the importance of natural constraints to agricultural land systems. In contrast to farm survey-based typologies (e.g., Weltin et al. 2017; Graskemper et al. 2021), our ALS typology provides spatially explicit insights into patterns of land cover, landscape structure, land-use intensity, climate and relief at the landscape scale. Thus, it offers valuable landscape-scale insights that contribute to a more comprehensive understanding of land-use patterns and their implications for farmland biodiversity.

As an unsupervised learning approach, cluster analysis does not rely on pre-defined classes. It enables learning by observation in contrast to learning by pre-structured knowledge as in supervised learning approaches such as random forest (Breiman 2001). Hence, the clustering approach adopted in this study is inverse to classification approaches based on expert-defined thresholds (Ellis and Ramankutty 2008; Rega et al. 2020; Dou et al. 2021). We refrained from using classification thresholds due to uncertainties associated with threshold definition. For example, there is evidence that increasing the amount of semi-natural habitats in agricultural landscapes has a positive effect on biodiversity (Sirami et al. 2019; Tscharntke et al. 2021). However, the semi-natural habitat cover needed to sustain farmland biodiversity and associated ecosystem services such as crop pollination and biological pest control depends on the landscape context. Proposed minimum semi-natural habitat cover ranges between 15% to more than 50% (Garibaldi et al. 2021; Eeraerts 2023), demonstrating uncertainties in the use of fixed thresholds to support farmland biodiversity.

### How the typology can inform and support the tailoring of Agri-Environmental Schemes

The characteristic patterns of indicator values of ALS types give insights into the ALS-specific pressures on farmland biodiversity, which can provide entry points for the selection and prioritization (i.e., tailoring) of conservation schemes such as AES. The significance of such a tailored approach to farmland biodiversity conservation is that by focusing on pressures that matter most in specific agricultural land systems, tailoring of AES is likely more effective than one-size-fits-all policies.

We use the characteristic combinations of indicator values at the cluster centres to identify ALS type-specific pressures and inform the AES tailoring focussing on combined aspects of land cover, landscape structure and land-use intensity. In particular, the indicators, whose values deviate strongly from the national average indicate outstanding conditions and, hence, key entry points for tailoring AES. Below, we illustrate how recurrent pressures indicated by the ALS typology can be used to tailor AES, drawing on examples from arable cash crop-dominated and animal husbandry-dominated ALS types.

Agricultural land system type A is dominated by the most intensive arable cash crop-farming in strongly simplified landscapes with the largest agricultural fields and highest share of arable land. These current pressures require a bundle of AES that address all three domains of land-use intensity, landscape structure and land cover. The intensive cash cropping entails high levels of pesticide and fertilizer inputs, which directly threatens farmland biodiversity (Kleijn et al. 2009; Geiger et al. 2010). To address these threats, AES should be prioritized that help to reduce agrochemical inputs. These include measures of integrated pest management (Vasileiadis et al. 2017), wide crop rotation, cultivation of pathogen-resistant cultivars (Saltzmann et al. 2024), among others. These measures should be combined with AES that improve the currently low landscape heterogeneity to further decrease negative biodiversity effects (Martin et al. 2019). For example, suitable AES encompass the reduction of field sizes (Sirami et al. 2019; Tscharntke et al. 2021) and implementation of landscape features such as field margins and buffer strips (Martin et al. 2019). Moreover, to move away from the arable land-dominated land cover, AES that support transitions from arable land to grassland can well complement the efforts to enhance farmland biodiversity (Török et al. 2011) in ALS type A.

Accordingly, ALS type B, which represents intensive arable farming with cash crops in moderately diverse landscapes, requires similar measures to address pressures accompanied with high levels of agro-chemical inputs. However, given that the indicators for landscape structure are close to the German-wide mean in ALS type B, lower priority should be given to AES that aim at increasing landscape heterogeneity.

Outstanding pressures on biodiversity in ALS type C regions arise from intensive pig and poultry farming complemented by intensively used arable land. The high livestock density in these regions lead to large amounts of slurry being applied to agricultural fields, resulting in high nitrogen inputs into neighbouring semi-natural ecosystems (de Vries et al. 2021). Nitrogen pollution has several negative consequences for biodiversity of terrestrial and freshwater ecosystems due to eutrophication, acidification, and exacerbation of other stresses (Bobbink et al. 2010; Dise et al. 2011). Hence, tailored AES are needed that help reduce livestock density and link it more closely to available agricultural land (Schulte-Uebbing and de Vries 2021). This aligns with targets of the European Green Deal’s Farm to Fork Strategy, which include reducing nutrient losses by 50% by 2030. These measures should be combined with AES that reduce agrochemical inputs in arable cash cropping by fostering integrated pest management (Vasileiadis et al. 2017), wider crop rotations, pathogen-resistant cultivars (Saltzmann et al. 2024) and transitions from arable land to grassland (Török et al. 2011).

ALS type D represents regions of highly intensive grassland systems, which are characterized by frequent cutting, leading to dominance of highly competitive species, and high nitrogen inputs due to high livestock densities and mineral fertilizers. Farmland biodiversity can be improved by reduced cutting frequencies (Potts et al. 2009), delayed mowing to protect meadow breeding birds (Breeuwer et al. 2009), favoring grazing over mowing (Busch et al. 2019), or abandonment of mineral fertilizers (Kleijn et al. 2009). Selecting and prioritizing these AES, combined with reducing livestock densities and improved manure management would aid transition to more heterogeneous grasslands and reduction of nitrogen losses (Schulte-Uebbing and de Vries 2021).

Because both ALS types C and D show indicator values for landscape structure that are close to the German-wide mean, lower priority should be given to measures that enhance landscape heterogeneity in these types. However, AES aimed at reducing land-use intensity with measures that maintain and improve existing hedgerows, field margins, or other features of high conservation value could further support biodiversity recovery.

ALS type G represents regions with extensive livestock farming in mountainous regions characterized by heterogeneous terrain, steep slopes, and short vegetation periods. Maintaining extensive livestock farming is desirable because extensive, small-scale mountain dairy farms retain high biodiversity meadows and pastures (MacDonald et al. 2000; Pornaro et al. 2021). However, due to the environmental conditions economic viable farming is challenging in these regions, which are risk of farmland abandonment leading to shrub encroachment and reforestation, which in turn leads to decrease in the diversity of rare and endemic species (MacDonald et al. 2000). Tailored AES in type G should therefore aim at maintaining low-intensity livestock farming and prevent landscape simplification due to land abandonment (Shipley et al. 2024). This should include result-based payment schemes that reward farmers for maintaining grasslands with high biodiversity without prescribing specific management (Wezel et al. 2018). Moreover, extensive grazing with domestic livestock and extensive mowing regimes should be re-established where they were abandoned.

Our ALS typology reveals particular pressure combinations helping to prioritize AES bundles that address multidimensional drivers of farmland biodiversity loss. Yet, the spatial resolution of our ALS typology entails uncertainty for tailoring AES to real-world conditions. Specifically, (i) natural boundaries of agricultural landscapes rarely align with grid cell borders, (ii) extend of farms are not confined within these grid cells, and (iii) AES are typically designed and implemented at the level of administrative units (e.g., districts, municipalities). These limitations could be overcome through a spatial aggregation, for instance, by summarizing all prevalent ALS types at the administrative unit level and prioritizing AES accordingly. This approach would enable farmers and decision makers to select from multiple AES that are both suitable for specific farm conditions and relevant administrative unit.

## Conclusions

We identified eight ALS types in Germany based on cluster analysis and expert consultation from the fields of agriculture, nature conservation, science, and administration. This typology synthesizes multidimensional associations and related interactions between land cover, landscape structure, land-use intensity of different production systems, climate, and relief. The ALS types could provide a sound basis for policy makers for tailoring conservation measures such as AES to effectively reduce pressures on farmland biodiversity.

The typology supports the systematic scaling and transfer of effective AES between regions with similar patterns of land cover, landscape structure, land-use intensity, and environmental conditions. Combining the ALS typology with insights from farmer typologies could further enhance the understanding of the farmers’ decision-making processes regarding the uptake of AES and help to further refine tailored AES to make them more appealing to farmers.

Future ALS classifications in Germany and Europe would profit from more detailed input data such as agricultural land-use data collected in the Integrated Administration and Control System (IACS), which is used to administer CAP payments to farmers in the EU (Leonhardt et al. 2023). Parcel and farm-specific data that are collected and administered by this system could greatly increase the spatial precision of variable cost estimation below the level of administrative units like municipalities. Moreover, IACS contains information on the implementation of AES by farmers (i.e., eco-schemes and agri-environment-climate measures). Future research should attempt to harness the potential of the IACS data for more comprehensively describing agricultural land systems and patterns of farmers’ uptake of AES at regional, national and European scale.

## Supplementary information

Supplementary information

## Data Availability

Indicators used to derive the typology of agricultural land systems and data representing the results are made available as tabular data and as geographically referenced data at the EDI data repository available at 10.6073/pasta/623f9bfa59b7802c5e07705170441a56.
