# Supplementary material for: Typology of Agricultural Land Systems in Germany to Support Tailoring of Agri-Environmental Schemes to Reduce Pressures on Farmland Biodiversity
Source: Environ Manage. 2026 Jun 22;76(7):222. doi: 10.1007/s00267-026-02490-5 (PMC13287224; doi:10.1007/s00267-026-02490-5)
Supplement: Supplementary file 1 — Supplementary information [file 267_2026_2490_MOESM1_ESM.docx]

**Supplemementary Material to the article: Typology of Agricultural Land Systems in Germany to Support Tailoring of Agri-Environmental Schemes to Reduce Pressures on Farmland Biodiversity**

Environmental Systems

https://doi.org/10.1007/s00267-026-02490-5

Martin Pingel^1*^, Diana Sietz^2^, Norbert Röder^3^, Sebastian Klimek^2^, Burkhard Golla^1^

^1^ Julius Kühn Institute (JKI) – Federal Research Centre for Cultivated Plants, Institute for Strategies and Technology Assessment, Kleinmachnow, Germany,

^2^ Thünen Institute of Biodiversity, Braunschweig, Germany

^3^ Thünen Institute of Rural Studies, Braunschweig, Germany

*corresponding author: [martin.pingel@julius-kuehn.de](mailto:martin.pingel@julius-kuehn.de)

# **Supplementary Tables**

**Supplementary Table 1**
Development of the set of indicators for the cluster analysis based on the initial set of indicators and the participation of experts. Farmland refers to land cover classes arable land, grassland, and permanent crops and horticulture.

| **Domain** | **Initial set of indicators** | **Concerns raised by experts** | **Alternative indicators suggested by experts** | **Authors final handling with the indicator** | **Justification** |
| --- | --- | --- | --- | --- | --- |
| **Land cover** | Arable land |  |  | Kept in the final indicator set |  |
|  | Special crops |  |  | Kept in the final indicator set |  |
|  | Grassland |  |  | Kept in the final indicator set |  |
|  | Forests | Dominant role of forest cover not appropriate for differentiation of agricultural land systems |  | kept in the final indicator set | Forests play a significant role in providing ecosystem services and are relevant in the context of land use change, particularly because of the high dynamism at the boundaries between agricultural land and forests (e. g., farmland abandonement). Also borders between agricultural land and forests provide habitats for ecoton species and, therefore are highly relevant for biodiversity. |
|  | Settlements |  |  | Kept in the final indicator set |  |
|  | Semi-natural habitats |  |  | kept in the final indicator set |  |
|  |  |  | Land cover of particular crop types like maize | Not included in the final indicator set | Crop type data have a high temporal variability, which is in conflict with the aim of attaining of a relatively stable typology. |
| **Landscape structure** | Shannon diversity of land cover classes |  |  | Kept in the final indicator set |  |
|  | Density of hedges | regional biases in coverage because of differences of survey methodology at the federal state level |  | removed from the final indicator set |  |
|  |  |  | Farmland edge density | included in the final indicator set | We agree with the experts that edge density is needed to reflect landscape configuration. |
|  |  |  | Farmland patch size | included in the final indicator set | We agree with the experts that patch sizeneeded to reflect landscape configuration. |
| **Land use intensity** | Variable costs for arable cash crops |  |  | Kept in the final indicator set |  |
|  | Variable costs for permanent crops and horticulture |  |  | kept in the final indicator set |  |
|  | Variable costs for pig and poultry farming |  |  | kept in the final indicator set |  |
|  | Variable costs for dairy farming and intensive beef fattening |  |  | Kept in the final indicator set |  |
|  | Variable costs for extensive livestock farming |  |  | Kept in the final indicator set |  |
|  |  |  | Livestock density | Not included in the final indicator set | High temporal variability of data, unsufficient data availability. Therefore, variable costs are used as it represents a more integrative indicator for land use, particularly in livestock based agricultural systems. |
| **Biophysical parameters** | Mean annual temperature |  |  | Kept in the final indicator set |  |
|  | Sum of annual precipitation |  |  | Removed from the final indicator set |  |
|  | Temperature seasonality |  |  | Kept in the final indicator set |  |
|  |  |  | Differentiation of climate data by months |  | A comprehensive data analysis of 76 climate parameters were done reveal indicators covering main German-wide gradients. These indicators were included in the final data set (see Supplementary Methods 2) |
|  | Relief heterogeneity |  |  |  |  |
|  |  |  | Soil type, soil quality | Not included in the final data set | Data on soil type are not numerical and continuous, which is a prerequisite for cluster analysis. Data on soil quality are not available at full coverage. See main mansuscript for further explanation. |

**Supplementary Table 2**Land cover classes obtained from the Basic Digital Landscape Model of the German Official Topographic Cartographic Information System of Germany (ATKIS Basic DLM) provided by the German Federal Agency for Cartography and Geodesy (BKG). Object numbers and attribute numbers refer to the ATKIS object catalogue (AdV 2008)

| **Land cover classes:**  **Coarse thematic resolution** | **Land cover classes:**  **Fine thematic resolution** | **Objects of ATKIS Basic DLM (German nomenclature and corresponding object numbers (*Objektart*) and attribute values (*Attributkennung und -Wert*)** |
| --- | --- | --- |
| Arable land | Arable land | *Ackerland (43001 VEG 1010)* |
| Special crops | Vineyards | *Rebfläche (43001 VEG 1040)* |
|  | Fruit orchards | *Obst- und Nussplantage (43001 VEG 1050),* |
|  | Orchard meadows  (extensive agroecosystems combining grassland and fruit trees) | *Streuobstwiese (43001 VEG 1021)*  *Streuobst auf Grünland ((54001 BWS 1900 auf 43001 VEG 1020)* |
|  | Horticulture | *Gartenbauland (43001 VEG 1030)* |
|  | Hops | *Hopfen (43001 VEG 1012)* |
|  | Treenurseries | *Baumschul (43001 VEG 1031)* |
| Grassland | Grassland | *Grünland (43001 VEG 1020)* |
| Forests | Forests | *Wald (43002)* |
| Settlements | Settlements | *Wohnbaufläche (41001)*  *Industrie- und Gewerbefläche (41002)*  *Halde (41003)*  *Fläche gemischter Nutzung (41006)*  *Fläche besonderer funktionaler Prägung (41007)*  *Sport-, Freizeit- und Erholungsfläche (41008)*  *Friedhof (41009)*  *Straßenverkehr (42001)*  *Platz (42009)*  *Bahnverkehr (42010)*  *Flugverkehr (42015)*  *Bauwerk oder Anlage für Industrie und Gewerbe (51002)*  *Hafen (52002)*  *Schleuse (52003)* |
| Seminatural habitats | Heaths | *Heide (43004)* |
|  | Bogs, Swamps and Wetlands | *Moor (43005)*  *Sumpf (43006)*  *Schilf, Röhricht (54001 BWS 1400)* |
|  | Small woody habitats | *Gehölz (43003)*  *Baumbestand, Gehölz oder Gebüsch auf Landwirtschaftliche Fläche (54001 BWS 1021, 1022, 1023, 1250, 1260 auf 43001)* |
|  | Wasteland  (non-cropped lands, wasteland, areas in successional stages, etc.) | *Unland (43007)* |

**Supplementary Table 3**Summary statistics of indicators that were used for cluster analysis. The following metrics are given: range (minimum – maximum), mean, standard deviation (SD), 25^th^, 50^th^, 75^th^, 95^th^, and 99^th^ percentile

| **Input variable** | **Unit** | **Range** | **Mean** | **SD** | **25^th^** | **50^th^** | **75^th^** | **95^th^** | **99^th^** |
| --- | --- | --- | --- | --- | --- | --- | --- | --- | --- |
| Arable land | % | 0 – 100 | 40 | 31 | 10 | 37 | 66 | 93 | 100 |
| Special crops | % | 0 – 100 | 1 | 6 | >1 | >1 | >1 | 07 | 33 |
| Grassland | %. | 0 – 100 | 18 | 19 | 4 | 12 | 26 | 60 | 85 |
| Forests | % | 0 – 100 | 25 | 27 | 1 | 13 | 3 | 84 | 93 |
| Settlements | % | 0 – 100 | 11 | 17 | >1 | 4 | 13 | 49 | 80 |
| Semi-natural habitats | % | 0 – 100 | 03 | 07 | >1 | 1 | 3 | 13 | 37 |
| Shannon diversity of land cover classes | - | 0 – 2.11 | 0.88 | 0.36 | 0.65 | 0.91 | 1.14 | 1.40 | 1.59 |
| Farmland edge density | m/ha | 0 – 723 | 111 | 60 | 66 | 107 | 151 | 216 | 266 |
| Farmland patch size | ha | <0.1 –148 | 16.5 | 24.5 | 4.0 | 7.5 | 17.0 | 64.9 | 148.0 |
| Variable costs for arable cash crops | EURO/ha | 0 – 1029 | 370 | 203 | 231 | 377 | 520 | 690 | 808 |
| Variable costs for permanent crops and horticulture | EURO/ha | 0 – 1024 | 48 | 140 | 0 | 6 | 35 | 197 | 1024 |
| Variable costs for pig and poultry farming | EURO/ha | 0 – 5633 | 530 | 1037 | 35 | 99 | 532 | 2896 | 5633 |
| Variable costs for dairy farming and intensive beef fattening | EURO/ha | 0 – 1857 | 562 | 420 | 249 | 472 | 770 | 1452 | 1857 |
| Variable costs for extensive livestock farming | EURO/ha | 0 – 671 | 87 | 112 | 28 | 46 | 98 | 314 | 671 |
| Mean annual temperature | °C | -0.7 – 11.8 | 9.5 | 0.9 | 9.1 | 9.6 | 10.0 | 10.8 | 11.3 |
| Temperature seasonality | °C range | 14.4 – 20.5 | 17.6 | 1.1 | 16.7 | 17.8 | 18.5 | 19.2 | 19.8 |
| Evapotranspiration March | mm | 20.3 – 47.1 | 36.5 | 3.50 | 34.2 | 36.6 | 38.8 | 42.3 | 45.5 |
| Relief heterogeneity | - | 0.1 – 573.0 | 25.6 | 31.4 | 4.7 | 14.8 | 36.0 | 81.7 | 139.0 |

**Supplementary Table 4**
Overview of grid cells that were excluded from the cluster analysis. The subsets of excluded grid cells can intersect; that means the cumulative sum is greater than the number of excluded grid cells

|  |  | **Number of grid cells (percentage of total number)** |
| --- | --- | --- |
| Total number of grid cells of the study area | | 361594 (100 %) |
| Subsets of grid cells that were excluded | Grid cells at national borders that covered less than 20% of the German territory | 1116 (> 1%) |
|  | Grid cells with missing data | 25504 (7 %) |
|  | excluded grid cells that contained less than 5% of land cover classes classified as “open” land cover (arable land, grassland, special crops, semi-natural habitats) | 39631 (11 %) |
| Number of grid cells included in the cluster analysis | | 315318 (87 %) |

**Supplementary Table 5**
Summary statistics of indicators by agricultural land system. The following metrics are given: mean, minimum, maximum, 25^th^, 50^th^, 75^th^percentile

| **Input Parameter** | **Unit** | **Statistic** | **Agricultural land system type** | | | | | | | | |
| --- | --- | --- | --- | --- | --- | --- | --- | --- | --- | --- | --- |
|  |  |  | **A** | **B** | **C** | **D** | **E** | **F** | **G** | **H** |  |
| Arable land | % | Mean | 84.80 | 65.31 | 59.06 | 24.32 | 34.09 | 14.60 | 5.23 | 18.55 |  |
|  |  | Min | 0.00 | 0.00 | 0.00 | 0.00 | 0.00 | 0.00 | 0.00 | 0.00 |  |
|  |  | Max | 100.0 | 100.0 | 100.0 | 100.0 | 92.70 | 69.40 | 88.29 | 70.60 |  |
|  |  | 25^th^ | 80.71 | 56.00 | 45.64 | 3.20 | 21.62 | 1.96 | 0.00 | 3.69 |  |
|  |  | 50^th^ | 89.97 | 66.92 | 62.19 | 17.71 | 34.44 | 10.49 | 0.03 | 15.11 |  |
|  |  | 75^th^ | 96.35 | 76.91 | 75.88 | 39.70 | 46.69 | 23.62 | 6.83 | 30.61 |  |
| Permanent crops and horticulture | % | Mean | 0.24 | 1.62 | 0.36 | 0.85 | 2.15 | 0.41 | 0.77 | 3.91 |  |
|  |  | Min | 0.00 | 0.00 | 0.00 | 0.00 | 0.00 | 0.00 | 0.00 | 0.00 |  |
|  |  | Max | 98.13 | 99.61 | 83.43 | 99.83 | 97.52 | 74.18 | 87.14 | 94.15 |  |
|  |  | 25^th^ | 0.00 | 0.00 | 0.00 | 0.00 | 0.00 | 0.00 | 0.00 | 0.00 |  |
|  |  | 50^th^ | 0.00 | 0.00 | 0.00 | 0.00 | 0.00 | 0.00 | 0.00 | 0.00 |  |
|  |  | 75^th^ | 0.00 | 0.00 | 0.00 | 0.00 | 0.48 | 0.00 | 0.00 | 1.38 |  |
| Grassland | % | Mean | 4.57 | 12.32 | 13.24 | 49.42 | 28.01 | 7.67 | 23.78 | 10.96 |  |
|  |  | Min | 0.00 | 0.00 | 0.00 | 0.00 | 0.00 | 0.00 | 0.00 | 0.00 |  |
|  |  | Max | 100.0 | 100.0 | 98.53 | 100.0 | 99.89 | 100.0 | 100.0 | 92.63 |  |
|  |  | 25^th^ | 0.00 | 3.86 | 4.45 | 29.71 | 17.37 | 1.25 | 11.09 | 3.01 |  |
|  |  | 50^th^ | 0.77 | 8.98 | 10.27 | 50.28 | 25.97 | 5.06 | 19.77 | 7.86 |  |
|  |  | 75^th^ | 4.09 | 16.40 | 18.68 | 69.11 | 36.81 | 10.33 | 32.76 | 15.46 |  |
| Forests | % | Mean | 4.59 | 9.97 | 12.68 | 8.76 | 22.65 | 69.79 | 60.70 | 10.57 |  |
|  |  | Min | 0.00 | 0.00 | 0.00 | 0.00 | 0.00 | 0.00 | 0.00 | 0.00 |  |
|  |  | Max | 65.19 | 62.22 | 94.91 | 82.73 | 69.54 | 95.00 | 94.99 | 67.69 |  |
|  |  | 25^th^ | 0.00 | 0.31 | 1.60 | 0.00 | 8.66 | 59.16 | 48.07 | 0.07 |  |
|  |  | 50^th^ | 0.46 | 4.37 | 6.25 | 2.30 | 21.16 | 72.22 | 62.35 | 3.91 |  |
|  |  | 75^th^ | 4.40 | 15.38 | 17.15 | 12.46 | 34.84 | 83.87 | 76.77 | 15.88 |  |
| Settlements | % | Mean | 2.94 | 6.91 | 10.44 | 9.35 | 9.44 | 2.17 | 5.35 | 45.92 |  |
|  |  | Min | 0.00 | 0.00 | 0.00 | 0.00 | 0.00 | 0.00 | 0.00 | 0.00 |  |
|  |  | Max | 94.04 | 60.42 | 95.00 | 94.81 | 76.41 | 93.94 | 94.28 | 95.00 |  |
|  |  | 25^th^ | 0.00 | 0.50 | 2.47 | 1.06 | 1.85 | 0.00 | 0.05 | 29.39 |  |
|  |  | 50^th^ | 0.55 | 3.64 | 5.76 | 4.47 | 5.95 | 0.08 | 1.34 | 44.27 |  |
|  |  | 75^th^ | 3.35 | 10.45 | 12.52 | 11.79 | 13.73 | 1.78 | 5.34 | 62.38 |  |
| Semi-natural habitats | % | Mean | 1.51 | 2.40 | 2.74 | 4.40 | 2.40 | 3.12 | 3.34 | 6.05 |  |
|  |  | Min | 0.00 | 0.00 | 0.00 | 0.00 | 0.00 | 0.00 | 0.00 | 0.00 |  |
|  |  | Max | 99.97 | 99.40 | 100.0 | 99.87 | 91.83 | 100.0 | 99.97 | 97.72 |  |
|  |  | 25^th^ | 0.00 | 0.00 | 0.16 | 0.17 | 0.00 | 0.00 | 0.00 | 1.06 |  |
|  |  | 50^th^ | 0.17 | 0.66 | 0.80 | 1.11 | 0.58 | 0.12 | 0.72 | 3.48 |  |
|  |  | 75^th^ | 1.31 | 2.57 | 2.32 | 3.83 | 2.69 | 2.01 | 2.79 | 7.72 |  |

| Shannon diversity of land cover classes | - | Mean | 0.388 | 0.854 | 0.914 | 0.896 | 1.194 | 0.710 | 0.828 | 1.106 |
| --- | --- | --- | --- | --- | --- | --- | --- | --- | --- | --- |
|  |  | Min | 0.000 | 0.000 | 0.000 | 0.000 | 0.008 | 0.000 | 0.000 | 0.200 |
|  |  | Max | 1.782 | 1.915 | 1.851 | 1.854 | 2.052 | 1.817 | 1.950 | 2.114 |
|  |  | 25^th^ | 0.162 | 0.700 | 0.729 | 0.707 | 1.076 | 0.514 | 0.643 | 0.920 |
|  |  | 50^th^ | 0.373 | 0.868 | 0.931 | 0.921 | 1.187 | 0.714 | 0.853 | 1.146 |
|  |  | 75^th^ | 0.578 | 1.023 | 1.111 | 1.108 | 1.310 | 0.891 | 1.029 | 1.326 |
| Farmland edge density | m/ha | Mean | 66.56 | 135.0 | 124.0 | 136.9 | 166.6 | 48.2 | 86.1 | 87.8 |
|  |  | Min | 0.000 | 0.850 | 0.030 | 0.040 | 9.890 | 0.010 | 0.090 | 0.020 |
|  |  | Max | 236.7 | 719.6 | 341.9 | 575.9 | 722.9 | 321.1 | 350.9 | 577.1 |
|  |  | 25^th^ | 48.4 | 101.5 | 99.42 | 104.5 | 134.2 | 26.75 | 53.80 | 47.33 |
|  |  | 50^th^ | 65.6 | 127.7 | 125.4 | 133.6 | 164.5 | 44.3 | 83.4 | 79.1 |
|  |  | 75^th^ | 83.60 | 160.7 | 150.5 | 165.9 | 196.2 | 65.7 | 112.5 | 115.4 |
| Farmland patch size | ha | Mean | 63.13 | 14.27 | 11.37 | 13.07 | 6.035 | 13.37 | 6.044 | 7.991 |
|  |  | Min | 0.058 | 0.480 | 0.195 | 0.384 | 0.498 | 0.000 | 0.055 | 0.000 |
|  |  | Max | 148.5 | 148.5 | 116.2 | 148.5 | 148.5 | 148.5 | 148.5 | 148.5 |
|  |  | 25^th^ | 32.98 | 6.14 | 6.68 | 6.21 | 3.08 | 3.41 | 2.20 | 2.68 |
|  |  | 50^th^ | 51.60 | 10.67 | 9.37 | 9.44 | 4.34 | 6.55 | 3.49 | 4.66 |
|  |  | 75^th^ | 84.22 | 18.65 | 13.50 | 15.22 | 6.49 | 15.34 | 5.92 | 8.95 |
| Variable costs for arable cash crops | Euro/ha | Mean | 522.0 | 508.3 | 480.0 | 139.2 | 303.1 | 382.4 | 115.7 | 422.9 |
|  |  | Min | 0.0 | 0.0 | 2.9 | 0.0 | 0.0 | 0.0 | 0.0 | 0.0 |
|  |  | Max | 1028.9 | 1028.9 | 1013.7 | 964.5 | 974.5 | 1028.9 | 798.2 | 1013.7 |
|  |  | 25^th^ | 412.32 | 410.65 | 389.32 | 41.35 | 221.51 | 282.55 | 8.08 | 299.44 |
|  |  | 50^th^ | 541.05 | 509.77 | 485.65 | 119.20 | 303.56 | 375.85 | 77.18 | 422.07 |
|  |  | 75^th^ | 633.37 | 604.15 | 569.45 | 210.60 | 386.84 | 477.83 | 198.25 | 546.58 |
| Variable costs for permanent crops and horticulture | Euro/ha | Mean | 24.214 | 64.008 | 38.185 | 20.254 | 37.131 | 44.247 | 22.037 | 135.36 |
|  |  | Min | 0.000 | 0.000 | 0.000 | 0.000 | 0.000 | 0.000 | 0.000 | 0.000 |
|  |  | Max | 1024.1 | 1024.1 | 1024.1 | 1024.1 | 10241 | 1024.1 | 1024.1 | 1024.1 |
|  |  | 25^th^ | 0.002 | 0.013 | 0.218 | 0.001 | 0.002 | 0.005 | 0.000 | 5.826 |
|  |  | 50^th^ | 1.102 | 12.735 | 24.735 | 0.433 | 3.442 | 6.889 | 0.004 | 34.165 |
|  |  | 75^th^ | 21.208 | 50.891 | 45.590 | 19.368 | 22.114 | 35.357 | 8.070 | 113.13 |
| Variable costs for pig and poultry farming | Euro/ha | Mean | 358.05 | 398.92 | 3301.8 | 259.19 | 289.96 | 360.90 | 112.72 | 308.46 |
|  |  | Min | 0.004 | 0.000 | 4.564 | 0.000 | 0.000 | 0.000 | 0.000 | 0.000 |
|  |  | Max | 5633.1 | 5633.1 | 5633.1 | 5633.1 | 5633.2 | 5633.1 | 5633.1 | 5633.1 |
|  |  | 25^th^ | 23.928 | 41.956 | 2034.7 | 19.493 | 42.117 | 34.307 | 16.993 | 31.521 |
|  |  | 50^th^ | 74.780 | 121.22 | 3434.5 | 83.403 | 99.466 | 92.723 | 50.314 | 88.120 |
|  |  | 75^th^ | 424.17 | 566.96 | 4686.4 | 234.24 | 292.30 | 467.17 | 101.06 | 286.01 |

| Variable costs for dairy farming and intensive beef fattening | Euro/ha | Mean | 322.50 | 343.67 | 773.06 | 1203.1 | 680.21 | 461.47 | 643.13 | 331.45 |
| --- | --- | --- | --- | --- | --- | --- | --- | --- | --- | --- |
|  |  | Min | 0.000 | 0.014 | 0.734 | 0.000 | 0.000 | 0.000 | 0.000 | 0.000 |
|  |  | Max | 1856.8 | 1856.8 | 1856.8 | 1856.8 | 1856.8 | 1856.8 | 1856.8 | 1856.8 |
|  |  | 25^th^ | 136.40 | 161.80 | 511.34 | 942.13 | 440.08 | 241.02 | 380.59 | 117.17 |
|  |  | 50^th^ | 274.17 | 307.80 | 672.47 | 1237.7 | 627.77 | 417.61 | 634.97 | 265.36 |
|  |  | 75^th^ | 441.66 | 471.78 | 985.79 | 1532.6 | 873.44 | 613.46 | 882.46 | 456.30 |
| Variable costs for extensive livestock farming | Euro/ha | Mean | 55.017 | 58.900 | 53.949 | 74.858 | 77.593 | 90.764 | 202.01 | 112.46 |
|  |  | Min | 0.000 | 0.000 | 0.013 | 0.003 | 0.000 | 0.000 | 0.000 | 0.000 |
|  |  | Max | 671.06 | 671.06 | 420.01 | 671.06 | 671.06 | 671.06 | 671.06 | 671.06 |
|  |  | 25^th^ | 14.336 | 20.774 | 33.120 | 30.243 | 29.625 | 28.654 | 60.010 | 35.325 |
|  |  | 50^th^ | 29.011 | 35.960 | 46.756 | 41.354 | 43.738 | 49.180 | 141.55 | 70.756 |
|  |  | 75^th^ | 61.648 | 68.189 | 68.070 | 63.570 | 88.499 | 107.52 | 272.00 | 141.08 |
| Mean annual temperature | °C | Mean | 9.663 | 9.689 | 10.263 | 9.544 | 9.170 | 9.481 | 8.268 | 10.194 |
|  |  | Min | 7.004 | 6.657 | 7.200 | 1.451 | 6.349 | 6.335 | -0.662 | 7.132 |
|  |  | Max | 11.570 | 11.639 | 11.517 | 11.485 | 11.627 | 11.644 | 11.403 | 11.831 |
|  |  | 25^th^ | 9.369 | 9.318 | 10.077 | 9.343 | 8.780 | 9.118 | 7.701 | 9.733 |
|  |  | 50^th^ | 9.620 | 9.628 | 10.267 | 9.697 | 9.152 | 9.516 | 8.409 | 10.153 |
|  |  | 75^th^ | 9.969 | 10.032 | 10.487 | 10.000 | 9.565 | 9.900 | 9.010 | 10.722 |
| Temperature seasonality | °C | Mean | 17.805 | 17.975 | 16.043 | 16.387 | 18.245 | 18.001 | 17.453 | 17.628 |
|  |  | Min | 14.495 | 14.888 | 14.780 | 14.457 | 15.282 | 14.791 | 14.423 | 14.457 |
|  |  | Max | 20.335 | 20.392 | 20.341 | 19.973 | 20.456 | 20.389 | 20.325 | 20.466 |
|  |  | 25^th^ | 17.165 | 17.280 | 15.775 | 15.755 | 17.690 | 17.316 | 16.823 | 16.726 |
|  |  | 50^th^ | 17.999 | 18.120 | 15.948 | 16.024 | 18.366 | 18.135 | 17.416 | 17.881 |
|  |  | 75^th^ | 18.493 | 18.624 | 16.207 | 16.618 | 18.801 | 18.705 | 18.046 | 18.466 |
| Evapo-transpiration March | mm | Mean | 34.767 | 37.118 | 37.072 | 34.511 | 37.536 | 36.470 | 34.512 | 39.125 |
|  |  | Min | 26.746 | 25.165 | 28.315 | 24.100 | 25.159 | 24.262 | 20.255 | 26.905 |
|  |  | Max | 46.858 | 47.060 | 45.390 | 45.640 | 47.002 | 47.039 | 45.994 | 47.038 |
|  |  | 25^th^ | 32.284 | 34.965 | 36.008 | 32.779 | 35.619 | 34.260 | 32.043 | 36.625 |
|  |  | 50^th^ | 35.139 | 36.941 | 37.015 | 34.281 | 38.035 | 36.555 | 34.501 | 39.010 |
|  |  | 75^th^ | 36.754 | 39.440 | 38.306 | 35.692 | 39.649 | 38.380 | 37.138 | 41.910 |
| Relief heterogeneity | - | Mean | 8.922 | 16.138 | 5.856 | 10.493 | 34.259 | 26.678 | 83.072 | 19.064 |
|  |  | Min | 0.093 | 0.176 | 0.266 | 0.077 | 0.288 | 0.126 | 0.218 | 0.354 |
|  |  | Max | 77.19 | 108.49 | 83.10 | 367.16 | 156.17 | 191.12 | 572.56 | 168.04 |
|  |  | 25^th^ | 3.761 | 4.907 | 1.861 | 1.563 | 19.897 | 6.198 | 52.777 | 4.675 |
|  |  | 50^th^ | 7.204 | 12.894 | 3.344 | 3.450 | 31.878 | 17.125 | 71.879 | 11.021 |
|  |  | 75^th^ | 12.026 | 23.958 | 6.652 | 11.057 | 46.426 | 39.702 | 97.155 | 26.747 |

# **Supplementary Figures**


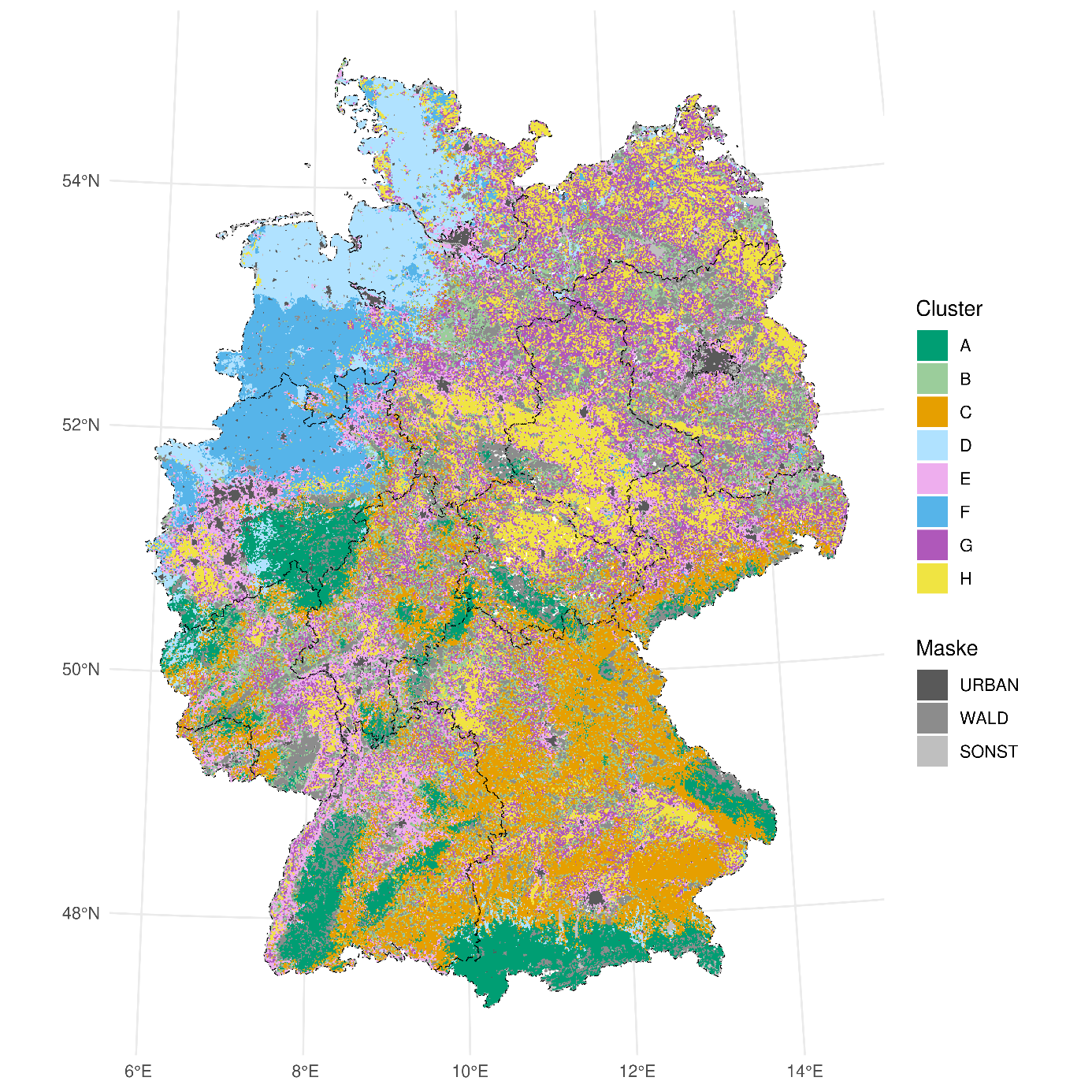


**Supplementary Fig. 1** Distribution of Clusters as first draft of agricultural land systems, which where presented to the experts at the work shop. Clusters result from k-means cluster analysis based on the initial set of indicators. Masked areas (grey-shaded) were not used for cluster analysis. Source of administrative borders: © GeoBasis-DE / BKG (2021)


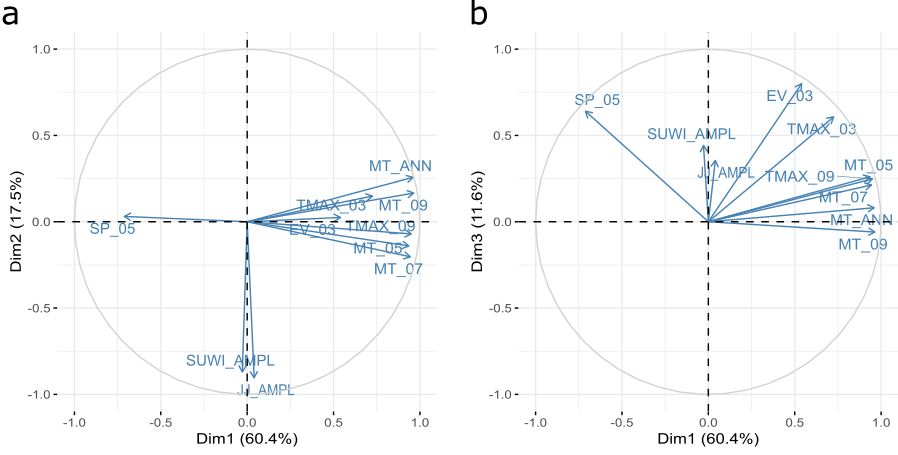


**Supplementary Fig. 2** Principal component analysis of climate variables. Ordination plots show first and second principal components (a), and first and third principal components (b). Only the climate variables with highest loadings on principal components 1-3 are shown. EV_03: potential evapotranspiration March, JJ_AMPL: temperature difference between of monthly means of July and January, MT_ANN: mean annual temperature, MT_05: mean temperature May, MT_07: mean temperature July, MT_09: mean temperature September, SP_05: Sum of precipitation May, SUWI_AMPL: temperature difference between of seasonal means of summer and winter, TMAX_03: maximum temperature March, TMAX_09: maximum temperature of September


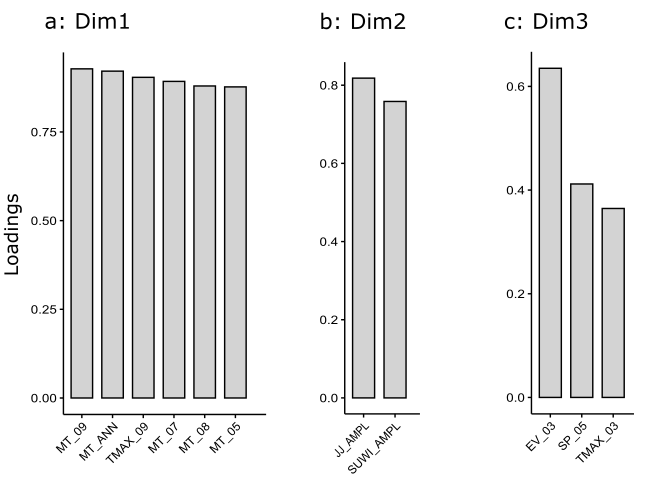


**Supplementary Fig. 3** Loadings of climate variables on first, second, and third principal component (a, b, and c, respectively). Loadings equal the squared coordinate of each climate variable on the respective principal component in the planar ordination plot (Fig. A1). Only the climate variables with highest loadings on principal components 1-3 are shown. EV_03: potential evapotranspiration March, JJ_AMPL: temperature difference between of monthly means of July and January, MT_ANN: mean annual temperature, MT_05: mean temperature May, MT_07: mean temperature July, MT_08: mean temperature August, MT_09: mean temperature September, SP_05: Sum of precipitation May, SUWI_AMPL: temperature difference between of seasonal means of summer and winter, TMAX_03: maximum temperature March, TMAX_09: maximum temperature of September


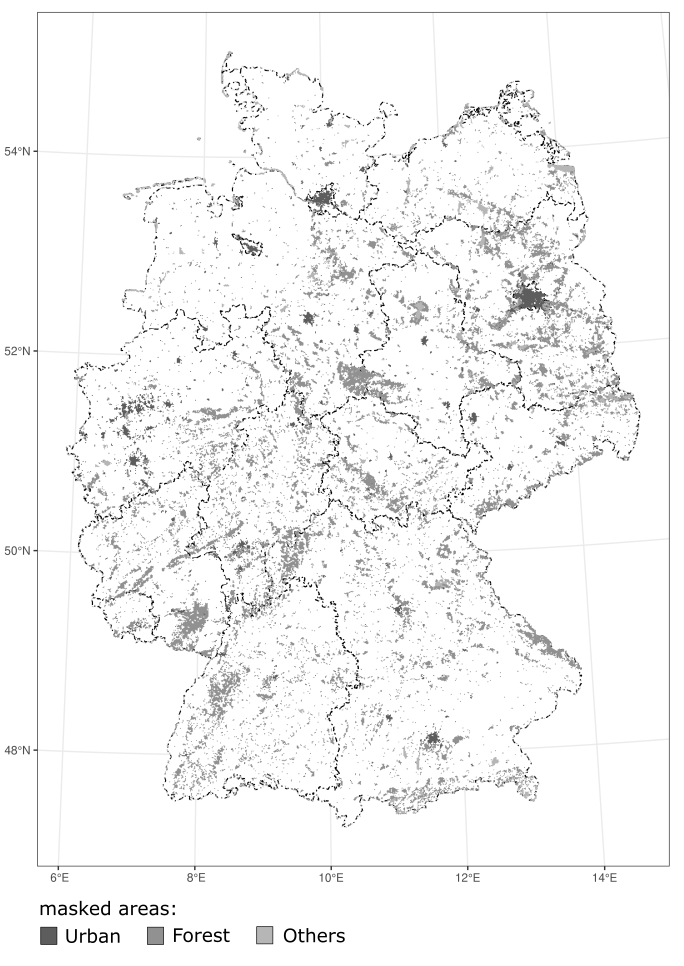


**Supplementary Fig. 4** Map of grid cells that were excluded prior to the cluster analyses. Excluded grid cells were classified by the proportion of land cover of settlements and forests as follows: Urban: > 50 % settlements, Forests: > 75 % Forests, Others: remaining grid cells. Dashed lines represent borders of German federal states (Source: © GeoBasis-DE / BKG (2021))

**
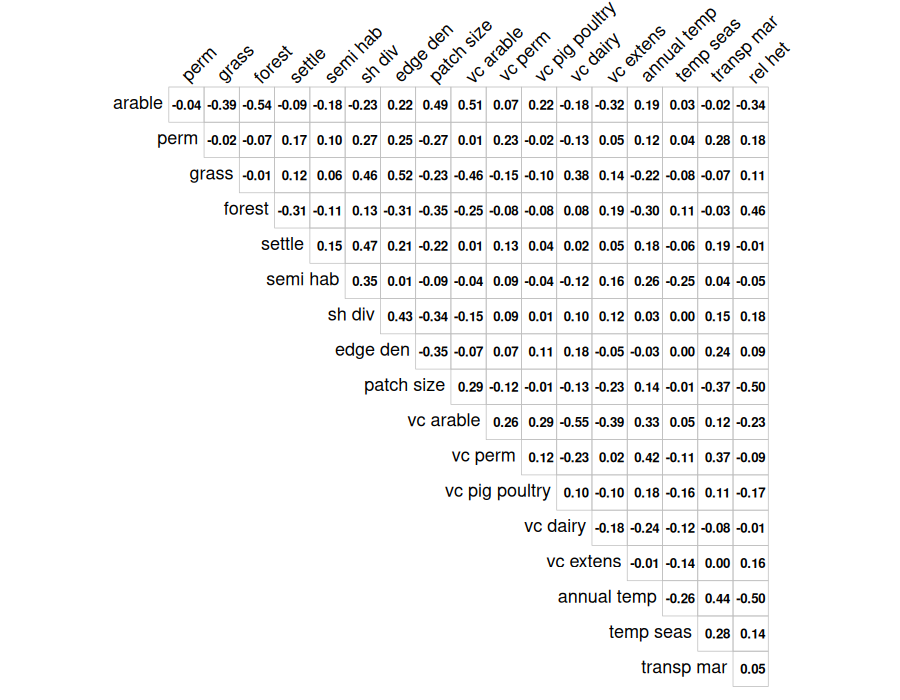
**

**Supplementary Fig. 5** Triangular matrix showing Spearman rank correlation coefficients between indicators. Pairwise correlation coefficients between indicators are given as percentage values for easier readability. Abbreviations: arable: arable land; perm: permanent crops and horticulture; grass: grassland; settle: settlements, semi hab: semi-natural habitats; sh div: Shannon diversity of land cover; edge den: farmland edge density; patch size: farmland patch size; vc arable: variable costs of arable cash crops; vc perm: variable costs of permanent crops and horticulture; vc pig poultry: variable costs of pig and poultry farming; vc diary: variable crops of dairy farming and beef fattening; vc extens: variable costs of extensive lifestock farming; annual temp: mean annual temperature; temp seas: temperature seasonality; transp mar: evapotranspiration in March, rel het: relief heterogeneity


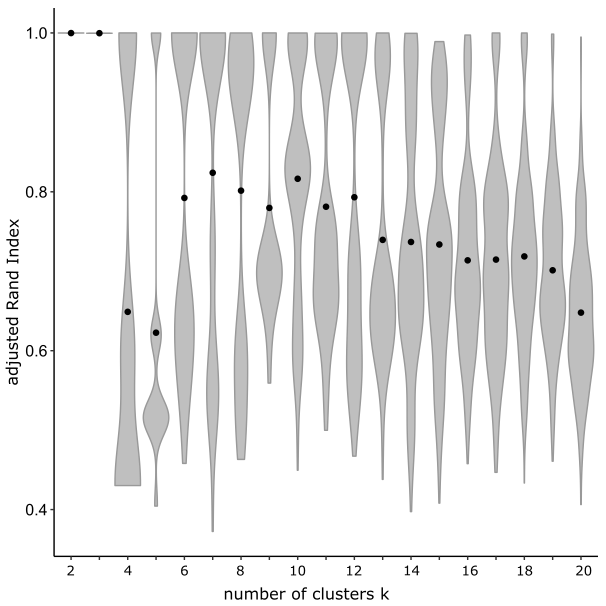


**Supplementary Fig. 6** Assessing optimal number of clusters (k) by calculation of stability of cluster results dependent on different cluster numbers. Adjusted Rand Index (aRI) was used for comparison of reference cluster results with 500 random cluster results for each pre-defined cluster number (k = 2 to 20). Violin plots represent distribution of aRI from 500 runs, black bullets represent mean


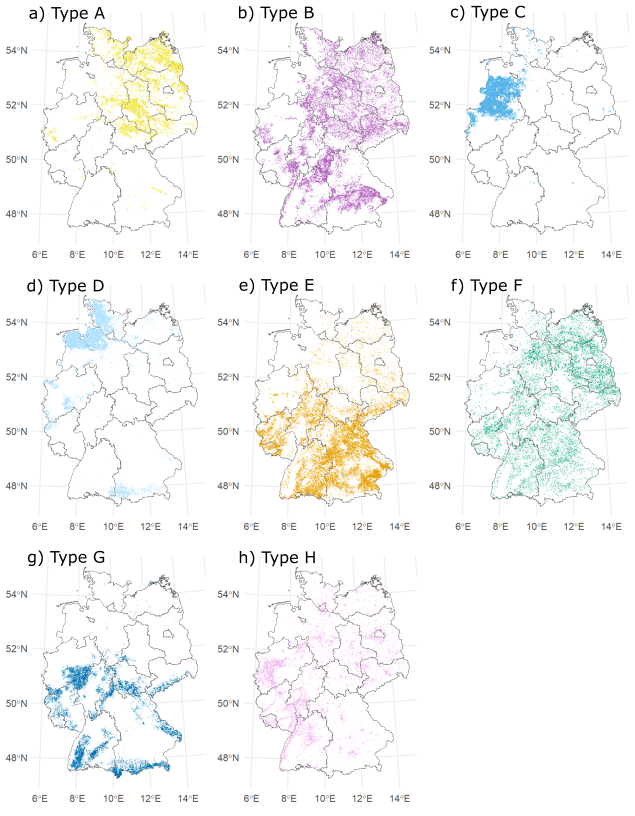


**Supplementary Fig. 7** Maps showing separated distribution of agricultural land system types A-H. The borders of German federal states are drawn in grey for better orientation (Source of administrative boarders: © GeoBasis-DE / BKG (2021)


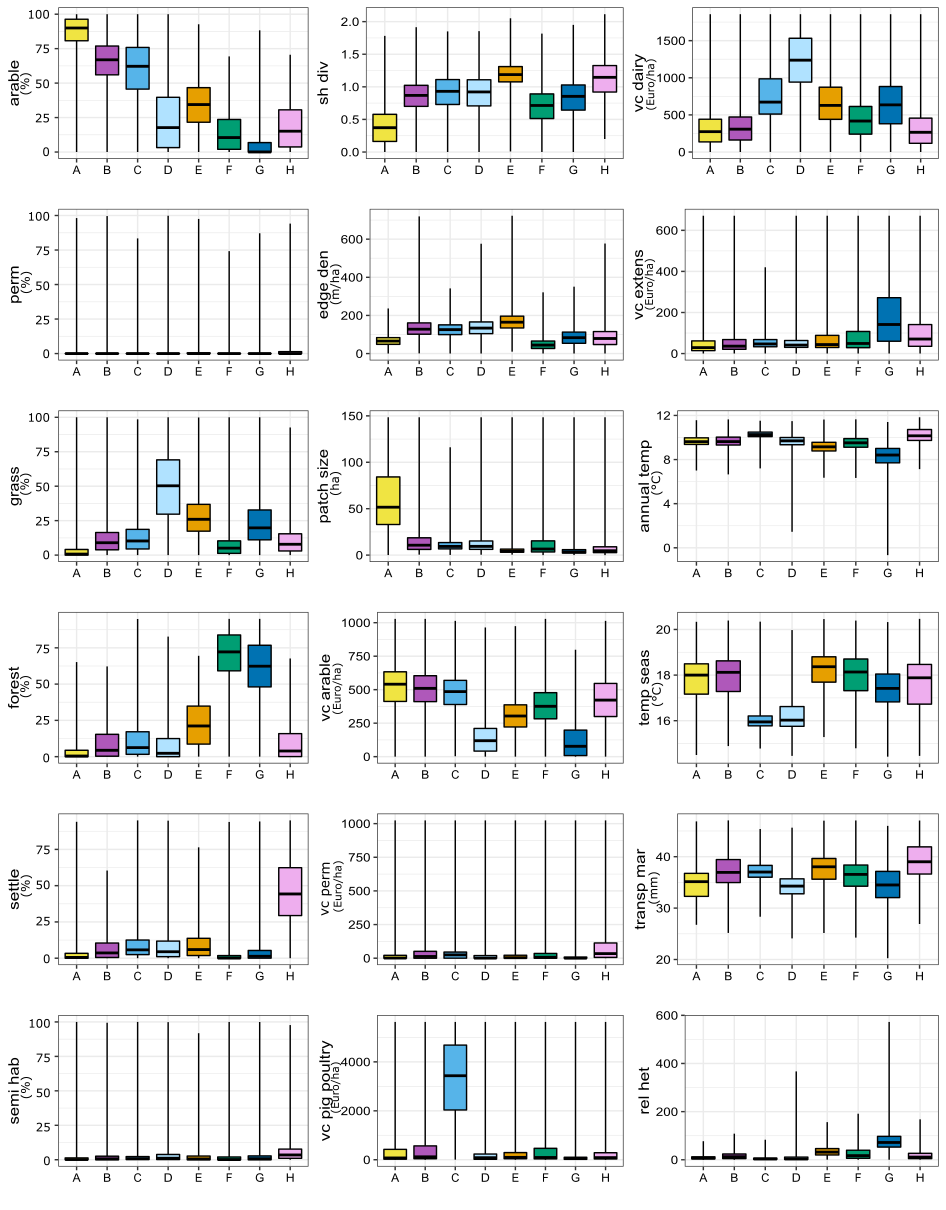


**Supplementary Fig. 8** Boxplots showing the distribution of each variable per agricultural land system type. Hinges indicate total range; boxes indicate medians and inter-quartile-ranges. Abbreviations: arable: arable land; perm: permanent crops and horticulture; grass: grassland; settle: settlements, semi hab: semi-natural habitats; sh div: Shannon diversity of land cover; edge den: farmland edge density; patch size: farmland patch size; vc arable: variable costs of arable cash crops; vc perm: variable costs of permanent crops and horticulture; vc pig poultry: variable costs of pig and poultry farming; vc diary: variable crops of dairy farming and beef fattening; vc extens: variable costs of extensive lifestock farming; annual temp: mean annual temperature; temp seas: temperature seasonality; transp mar: evapotranspiration in March, rel het: relief heterogeneity

# **Supplementary Methods 1**

**Agro-economic perspectives on the use of variable costs as indicator for land-use intensity**

The use of variable costs as indicator for land-use intensity allowed us to put land-use intensity into the context of agro-economic scenarios because it is linked to natural and monetary yields. When deciding on adoption of conservation measures, e.g., as provided by the CAP, farmers need to consider consequences for their farm economic viability balancing the income from subsidies for adopting conservation measures against opportunity costs. Farms and areas with low variable costs per hectare are associated with relatively low market output per hectare. Here, the relative economic importance of subsidies is higher (Haß et al. 2022, p. 37) and, hence, more relevant for maintaining economic viability of the farm. These farms and regions are more likely to adopt voluntary agri-environmental measures for farmland biodiversity compared to more intensively managed farms and regions (Lastra-Bravo et al. 2015; Wąs et al. 2021).

The differentiation of variable costs by production system further supports the interpretation of entrepreneurial decisions of farmers. Depending on the production system (e.g., cropping systems versus livestock systems), farmers have different time horizons to consider in their strategic decisions: Within arable (annual) cash crops systems, the decision which crops to grow is largely determined on a short-term, even annual, decision. Generally, the used machinery and equipment is usable for treatment and harvest of several annual crop types, and bulk commodities are produced, which are relatively easy to trade. In contrast, the decision which crop to grow is a long-term decision for permanent crops as these crops need several years before they reach peak-production and also demand specialized machinery, know-how and distribution channels. The same applies to most livestock production systems, because investments in barn conversions, for example, to meet higher standards of animal welfare, take many years to pay off.

**Additional information on input categories of livestock production systems**

Pig and poultry farming is at present only indirectly linked to the actual land-use as in most cases the animals are kept in housing systems year-round and fodder supply by imports have a great importance. However, for reasons associated to income taxation and ordinance law (e.g., nitrates directive) certain stocking limits must not be exceeded on farm level.

In intensive dairy farming and intensive beef fattening systems, the vast majority of animals is also kept in-house year-round. For these systems, silage corn is an important roughage feedstock. We included silage corn production for biogas fermenters here, as the on-field production system is nearly indistinguishable whether the silage is produced as feedstock for biogas fermenters or to feed dairy cattle and fattening bulls.

Extensive livestock farming system contains different types of grazing livestock, that are all fed mainly be grass or grass conserves and in which grazing plays an important role for the fodder provision during the vegetation period.

# Supplementary Methods 2

**Derivation of climate indicators**

Because climate variables often show high multi-collinearity, including a lot of indicators for climate would severely bias the cluster analysis by overweighing climate without knowledge of the underlying climate gradients driving the cluster analysis. Therefore, we aimed to reduce the dataset to three non-correlated indicators representing the main gradients of climate in Germany. These should be easy to interpret and implement in future updates of the typology of agricultural land systems.

In the first step, we collected and calculated 76 climate parameters using the 1 km²-grid based data from the Climate Data Center of the German Weather Service (DWD 2022):

- monthly mean of daily temperature,
- annual mean of daily temperature,
- temperature difference between of monthly means of July and January,
- temperature difference between of seasonal means of summer and winter,
- monthly mean of daily minimum temperature,
- monthly mean of daily maximum temperature,
- monthly total precipitation
- annual total precipitation
- monthly accumulated potential evapotranspiration over grass
- monthly soil moisture under grass and sandy loam

Data were averaged over the period from 2000 to 2019 and resampled to match the hexagonal grid used for the typology.

Next, we conducted a Principal Component Analysis (PCA) using all grid cells and climate variables. Principal component analysis and visualisations were done using the R-packages FactoMinerR version 2.4 (Lê et al. 2008) and factoextra version 10.0.7 (Kassambara and Mundt 2020).

Supplementary Figure 2 shows the PCA with Dimensions 1 and 2 (a), and 1 and 3 (b). The first three axes cover 90.1 % of explained variation in the data. The squared coordinates of variables on the axis of interests were interpreted as the loadings of variables (Supplementary Fig. 2).

The first axis was positively correlated with temperature variables (Supplementary Fig. 2a). The five parameters with the highest contribution to the variance of the first component were in decreasing order: mean temperature of September, mean annual temperature, maximum temperature of September, mean temperature of July, mean temperature of August. The squared coordinate values for these variables ranged between 0.88 and 0.93 (Supplementary Fig. 3a). Additionally, there is a high collinearity between these variables. Because many variables contributed almost equally to the first component, we decided to choose mean annual temperature as indicator for clustering. Acknowledging that other variables were equally (or even a bit more important), mean annual temperature has the advantage that it is easy to interpret and to communicate in the context of environmental change.

The variables ’temperature difference between of monthly means of July and January’ and ’temperature difference between of seasonal means of summer and winter’ showed the highest loading on the second principal component (Supplementary Figs. 2a and 3b). Both variables express the seasonality of temperature and are interpretable in the context of the atlantic-continental climate gradient, which shapes the European climate from West to East. Since the squared coordinate values were slightly higher for the July-January difference than for the Summer-Winter difference (0.82 vs 0.76), we chose the former as indicator for the cluster analysis.

For the third axis (Supplementary Figs. 2b and 3c), ‘potential evapotranspiration in March’ showed the highest squared coordinate value (0.63), and ‘precipitation in March showed the second highest value’ (0.41). Both variables were only weakly correlated (Spearman’s correlation coefficient = 0.31), but both represent an aspect of rain supply and water consumption of the vegetation during spring. Evapotranspiration is a modelled variable, which considers many factors including temperature and precipitation (DWD Climate Data Center 2018). In the context of agriculture, this variable may be more appropriate to describe the water regime for cultivated plants. Further, it may indicate the risk of spring droughts. Therefore, potential evapotranspiration in March was used as third climate indicator for cluster analysis.

Supplementary References

Arbeitsgemeinschaft der Vermessungsverwaltungen der Länder der Bundesrepublik Deutschland (2008) ATKIS-Objektartenkatalog Basis-DLM (7.1.0). Dokumentation zur Modellierung der Geoinformationen des amtlichen Vermessungswesens:. https://www.adv-online.de/GeoInfoDok/GeoInfoDok-6.0/. Accessed 3 January 2022. Accessed 3 January 2022

DWD (2022) DWD Climate Data Center (CDC). https://opendata.dwd.de/. Accessed 3 January 2022

DWD Climate Data Center (2018) Monthly grids of the accumulated potential evapotranspiration over grass, version 0.x. https://opendata.dwd.de/climate_environment/CDC/grids_germany/monthly/evapo_p/DESCRIPTION_gridsgermany_monthly_evapo_p_en.pdf. Accessed 27 March 2025

Haß M, Deblitz C, Freund F, Kreins P, Laquai V, Offermann F, Pelikan J, Sturm V, Wegmann J, Witte Td, Wüstemann F, Zinnbauer M (2022) Thünen-Baseline 2022 - 2032: Agrarökonomische Projektionen für Deutschland. Thünen Report, vol 100. Johann Heinrich von Thünen-Institut, Braunschweig. https://doi.org/10.3220/REP1667811151000

Kassambara A, Mundt F (2020) factoextra: Extract and Visualize the Results of Multivariate Data Analyses. https://CRAN.R-project.org/package=factoextra

Lastra-Bravo XB, Hubbard C, Garrod G, Tolón-Becerra A (2015) What drives farmers’ participation in EU agri-environmental schemes?: Results from a qualitative meta-analysis. Environmental Science & Policy 54:1–9. https://doi.org/10.1016/j.envsci.2015.06.002

Lê S, Josse J, Husson F (2008) FactoMineR : An R Package for Multivariate Analysis. J. Stat. Soft. 25. https://doi.org/10.18637/jss.v025.i01

Wąs A, Malak-Rawlikowska A, Zavalloni M, Viaggi D, Kobus P, Sulewski P (2021) In search of factors determining the participation of farmers in agri-environmental schemes – Does only money matter in Poland? Land Use Policy 101:105190. https://doi.org/10.1016/j.landusepol.2020.105190
